# Supplementary material for: Pyrolysis Kinetic Study of Polylactic Acid
Source: Polymers (Basel). 2022 Dec 20;15(1):12. doi: 10.3390/polym15010012 (PMC9823905; doi:10.3390/polym15010012)
Supplement: Supplementary file 1 [file polymers-15-00012-s001.zip › polymers-2098558-supplementary.pdf]

polymers

MDPI

Table S1. List of the most used solid-state reaction models ($f(\alpha)$). ((Mumbach et al. (2019) [1], Aboulkas et al. (2010) [2]).

Model	Mechanism	n
1. *Reaction-Order Models:*		
	First-order reaction (F1)	1
$f(\alpha) = (1 - \alpha)^n$	Second-order reaction (F2)	2
	Third-order reaction (F3)	3
2. *Diffusion Models:*		
$f(\alpha) = 1/(2\,\alpha)$	One-dimensional diffusion (D1)	
$f(\alpha) = 1/[-ln\,(1 - \alpha)]$	Two-dimensional diffusion (D2)	NA
$f(\alpha) = 3/(2[1 - (1 - \alpha)^{1/3}])$	Three-dimensional diffusion (D3)	
3. *Avrami-Erofeev Models:*		
	A2	2
$f(\alpha) = n(1 - \alpha)[-ln(1 - \alpha)]^{(n-1)/n}$	A3	3
	A4	4
4. *Power-Law Models:*		
	P2	2
$f(\alpha) = n\,\alpha^{(n-1)/n}$	P3	3
	P4	4
5. *Geometrical Contraction Models:*		
	Prout-Tompkins (R1)	1
$f(\alpha) = n\,(1 - \alpha)^{(n-1)/n}$	Contracting cylinder (R2)	2
	Contracting sphere (R3)	3

1. Mumbach, G.D.; Alves, J.L.F.; Silva, J.C.G.D.; Sena, R.F.D.; Marangoni, C.; Machado, R.A.F.; Bolzan, A. Thermal investigation of plastic solid waste pyrolysis via the deconvolution technique using the asymmetric double sigmoidal function: Determination of the kinetic triplet, thermodynamic parameters, thermal lifetime and pyrolytic oil composition for clean energy recovery. *Energy Convers. Manag.* **2019**, *200*, 112031. https://doi.org/10.1016/j.enconman.2019.112031.
2. Aboulkas, A.; El Harfi, K.; El Bouadili, A. Thermal degradation behaviors of polyethylene and polypropylene. Part I Pyrolysis kinetics and mechanisms. *Energy Convers. Manag.* **2010**, *51*, 1363–1369. https://doi.org/10.1016/j.enconman.2009.12.017.
